# Response to the comment by Dr Yarlas

**DOI:** 10.1111/eci.13652

**Published:** 2021-07-28

**Authors:** Alberto Aimo

**Affiliations:** ^1^ Institute of Life Sciences Scuola Superiore Sant’Anna Pisa Italy; ^2^ Cardiology Division Fondazione Toscana Gabriele Monasterio Pisa Italy

## CONFLICTS OF INTEREST

None.

Dear Editor,

Amyloid transthyretin (ATTR) amyloidosis is caused by the systemic deposition of transthyretin molecules, either normal (wild‐type ATTR, ATTRwt) or mutated (variant ATTR, ATTRv). ATTR amyloidosis is a progressive disease with a severe impact on patient quality of life (QoL)^1^, ^2^, ^3^. Nonetheless, limited attention has been paid to QoL so far, and no specific tools for QoL assessment in ATTR amyloidosis currently exist^3^. QoL can be evaluated through patient‐reported outcome measures, which are completed by patients, or through scales, which are compiled by clinicians. The scales investigate QoL either directly or indirectly, that is by assessing the degree of functional impairment and limitations imposed by the disease. In a recent review article, we performed a systematic assessment of the measures of QoL evaluated in phase 2 and phase 3 clinical trials on ATTR amyloidosis^3^. We are grateful to Dr Yarlas from QualityMetric for pointing out that we referred only to the 36‐Item Short Form Survey (SF‐36) instead of mentioning specifically its second version.

The 36‐Item Short Form Health Survey questionnaire (SF‐36) is a very popular tool to assess health‐related QoL. Indeed, a PubMed search for ‘SF‐36 health survey’ on 11 July 2021 yielded 13,715 results. The SF‐36 was designed as a generic indicator of health status to be used in population surveys and in studies evaluating health policies. It can also be used as an outcome measure in clinical practice and research. The concept of the SF‐36 was devised in the 1970s, and an 18‐item scale was developed in 1984, followed by a 20‐item form (SF‐20) in 1986. The SF‐36 was constructed to overcome limitations in the SF‐20. Version 1 of the SF‐36 was introduced in 1996. Version 2 of the SF‐36 changed some dichotomous answers to 5‐point scales and slightly altered the wording of several items. The development of the SF‐36 is now coordinated by QualityMetric^4^.

As a generic instrument, the SF‐36 was designed to be applicable to a wide range of types and severities of conditions. It includes multi‐item scales to measure the following 8 dimensions: physical functioning, role limitations due to physical health problems, bodily pain, social functioning, general mental health, role limitations due to emotional problems, vitality, energy or fatigue and general health perceptions. Additionally, a question covers change in health status over the past year.

As for the ‘additional error’ pointed out by Dr Yarlas, the correct reference regarding the polyneuropathy disability (PND) score was the paper by Dyck et al^5^, 2019. The extent of disability in ATTRv amyloidosis has been typically evaluated through the familial amyloidotic polyneuropathy (FAP) staging system and/or the PND scoring system. FAP staging was developed in an endemic area of Portugal^6^ in 1980. FAP stage 1 is defined by unassisted walking, in which patients typically experience mild bilateral neuropathy in the feet and legs; stage 2 is defined by the patient requiring assistance walking with crutches or sticks; stage 3 is defined by the patient becoming wheelchair‐bound or bedridden because of severe neuropathy. PND scoring involves a greater separation of disease stages: a score of I indicates sensory disturbance with preserved walking capacity; II indicates difficult, but unassisted walking; IIIa indicates the need of one stick or crutch for walking; IIIb indicates the requirement of 2 sticks or crutches for walking; and IV denotes a patient wheelchair‐bound or bedridden^7^. Assessing ATTRv polyneuropathy in this manner can provide a broad indication of the global disease state, but transition from one FAP stage or PND score to another can take up to 5 years^7^. These metrics are then poorly effective to track disease progression over shorter time periods and cannot be proposed as outcome measures in clinical trials, where the Neuropathy Impairment Score (NIS), the NIS‐lower limb (NIS‐LL; a subset of the NIS) and the NIS+7 (a variation of NIS that includes nerve conduction studies and quantitative sensory and autonomic endpoints) have been used instead ^5^.

Despite the imprecisions carefully highlighted by Dr Yarlas, the main message of our review remains, which is that there are no specific measures to assess QoL in patients with ATTR amyloidosis, not even the SF‐36v2^®^ Health Survey by QualityMetric. The challenge for clinicians, researchers and private companies is to develop such metrics to improve the management of these patients.
